# Second Law and Non-Equilibrium Entropy of Schottky Systems—Doubts and Verification–

**DOI:** 10.3390/e20100740

**Published:** 2018-09-28

**Authors:** Wolfgang Muschik

**Affiliations:** Institut für Theoretische Physik, Technische Universität Berlin, 10623 Berlin, Germany; muschik@physik.tu-berlin.de

**Keywords:** non-equilibrium entropy, schottky systems, inert partition, second law, contact temperature, entropy-free thermodynamics, defining inequalities, adiabatical uniqueness, clausius inequality of open systems

## Abstract

Meixner’s historical remark in 1969 “... it can be shown that the concept of entropy in the absence of equilibrium is in fact not only questionable but that it cannot even be defined....” is investigated from today’s insight. Several statements—such as the three laws of phenomenological thermodynamics, the embedding theorem and the adiabatical uniqueness—are used to get rid of non-equilibrium entropy as a primitive concept. In this framework, Clausius inequality of open systems can be derived by use of the defining inequalities which establish the non-equilibrium quantities contact temperature and non-equilibrium molar entropy which allow to describe the interaction between the Schottky system and its controlling equilibrium environment.

## 1. Introduction

The Second Law (SL) has many faces: there are different formulations in phenomenological thermodynamics, statistics, kinetics and quantum theory. Here, only phenomenological considerations are made in the range of Schottky systems [1] that are discrete systems which can exchange heat, power and material with their environment by suitable partitions. The field formulation of thermodynamics is as well as a historical survey and an axiomatic treatment out of scope.

Historically, the Second Law launches with two verbal formulations concerning irreversible cyclic processes of discrete systems: the principle of Kelvin [2] and that of Clausius [3] which are so well known that they can be formulated in a short form [4]:

Kelvin: There is no Thomson process (but friction processes exist),
Clausius: There is no Clausius process (but heat conduction processes exist).
Accepting additionally
Carnot [5]: Reversible Carnot processes exist (not really, but as a mathematical closure of irreversible processes),

and starting with these verbal statements of Kelvin, Clausius and Carnot, the following Clausius inequality valid for cyclic processes in closed systems can be derived [6] in an up-to-date formulation

(1) $$\oint \frac{\overset{•}{Q}\left(t\right)}{T^{□}\left(t\right)}dt\;\leq \;0.$$

Here, $\overset{•}{Q}$ is the heat exchange per time between the controlling heat reservoir and the Schottky system (to be defined below) which can be measured by calorimetry. $T^{□}$ is the thermostatic (equilibrium) temperature of the heat reservoir which controls the cyclic process.

For more detailed understanding of the Clausius inequality, the following questions arise and have to be discussed below

1. What is the meaning of the parameter *t* in connection with the < and = signs in Clausius inequality?
2. On what state space characterizing the system does the cyclic process operate?
3. How can the inequality be extended to open systems?
4. What are the relationships between Clausius inequality and entropies?

Beyond these questions, a shortcoming of the derivation of Clausius inequality (1) has to be taken into consideration: the statement of Carnot claims the existence of reversible processes, a presupposition which should not be used here, because the physical meaning of reversible processes is not evident and has to be defined properly in the course of this paper. Additionally, the Carnot theorem of reversible Carnot processes

(2) $$\frac{{\overset{•}{Q}}_{1}\Delta_{1}t}{T_{1}^{□}}+\frac{{\overset{•}{Q}}_{2}\Delta_{2}t}{T_{2}^{□}}\;=\;\frac{Q_{1}}{T_{1}^{□}}+\frac{Q_{2}}{T_{2}^{□}}\;=\;0$$

which is used in the derivation of (1) is a special case of this relation which should be proved. Consequently, the verbal formulation of the Second Law—statements Kelvin and Clausius—cannot be transformed by the statement Carnot into Clausius inequality without a logical fallacy. A remedy may be to set (1) as an axiom, or better, to derive it in connexion with a suitable definition of a non-equilibrium entropy, a way which is worked out here serving as a motivation to have a look at this “antiquated stuff” again.

The starting point is that the sign of the left-hand side of (1) is unknown. Using this fact, the third question can be taken into attac by investigating the following expression

(3) $$SL\;:=\;\oint \left(\frac{\overset{•}{Q}\left(t\right)}{T^{□}\left(t\right)}+s^{□}\cdot {\overset{•}{n}}^{e}\right)dt.$$

Here, ${\overset{•}{n}}^{e}$ is the mole numbers exchange between the controlling reservoir and the system, and $s^{□}$ is the molar equilibrium entropy of this reservoir. SL has a characteristic shape: $\overset{•}{Q}$ and ${\overset{•}{n}}^{e}$ are exchange quantities referring to the system, whereas $T^{□}$ and $s^{□}$ belong to the controlling reservoir which generates the cyclic process of the system.

Although still in use today, Clausius inequality is a historical relation. In the meantime, there are “as many formulations of the Second Law as there are authors” [7]. Some of these formulations can be found in [8,9,10,11,12,13,14].

## 2. Schottky Systems

### 2.1. Exchanges and Partitions

A system G, described as undecomposed and homogeneous, which is separated by a partition ∂G from its environment $G^{□}$ is called a Schottky system [1], if the interaction between G and $G^{□}$ through ∂G can be described by

(4) $$\text{heat exchange}\;\overset{•}{Q},\;\text{power exchange}\;\overset{•}{W},\;\text{and material exchange}\;{\overset{•}{n}}^{e}.$$

The power exchange is related to the work variables ***a*** of the system

(5) $$\overset{•}{W}\;=\;A\cdot \overset{•}{a}.$$

Here, A are the generalized forces which are as well known as the work variables. Kinetic and potential energy are constant and therefore out of scope. $\overset{•}{Q}$ is measurable by calorimetry and the time rate of the mole numbers due to material exchange ${\overset{•}{n}}^{e}$ by weigh.

Using the exchange quantities, partitions ∂G of different properties can be defined: If by choice of an arbitrary environment $G^{□}$, the following exchange quantities vanish identically

(6) $$\begin{array}{ccc} \overset{•}{W}\;\equiv \;0 & ⟶ & \text{power-isolating}, \end{array}$$

(7) $$\begin{array}{ccc} {\overset{•}{n}}^{e}\;\equiv \;0 & ⟶ & \text{material-isolating}, \end{array}$$

(8) $$\begin{array}{ccc} \overset{•}{Q}\;\equiv \;0\;\wedge \;{\overset{•}{n}}^{e}\;\equiv \;0 & ⟶ & \text{adiabatic}, \end{array}$$

(9) $$\begin{array}{ccc} \text{adiabatic and power-isolating} & ⟶ & \text{isolating}, \end{array}$$

the partition is called ⟶⊠. A system is called thermally homogeneous, if it does not contain any adiabatic partition in its interior.

An inert partition does not absorb or emit heat, power and material [15]. It is defined by the following equations [16,17]

(10) $$\overset{•}{Q}\;=\;-\overset{•}{Q}\;{}^{□},\;A\cdot \overset{•}{a}\;=\;A^{□}\cdot \overset{•}{a},\;{\overset{•}{n}}^{e}\;=\;-\overset{•}{n}\;{}^{e□}.$$

Here, the ${}^{□}$-quantities belong to the system’s controlling environment $G^{□}$. The work done on the system is performed by the environment using its generalised forces $A^{□}$ and orientated at the work variables of the system. The permeability of ∂G to power and material is described by (10)${}_{2,3}$.

### 2.2. State Spaces and Processes

The cyclic process SL in (3) is defined on a state space because otherwise the path integral makes no sense. Here, a large state space Z is used [18] which is decomposed into its equilibrium subspace and the non-equilibrium part

(11) $$Z\;=\;(z_{eq},z_{ne})\;\in \;Z.$$

Here, states of equilibrium $z_{eq}$ are defined by time independent state of an isolated Schottky system. The equilibrium subspace is too small for decribing non-equilibrium. Consequently, it has to be extended by the non-equilibrium part $z_{ne}$ of Z. If the considered system is in equilibrium, the non-equilibrium variables become dependent on the equilibrium ones

(12) $$Z^{eq}\;=\;\left(z_{eq},z_{ne}\left(z_{eq}\right)\right)\;\in \;Z^{eq}.$$

The variables of the equilibrium subspace are determined by the Zeroth Law: The state space of a thermally homogeneous Schottky system in equiIibrium is spanned by the work variables, the mole numbers and the internal energy

(13) $$z_{eq}\;=\;\left(a,n,U\right)\;⟶\;Z\;=\;\left(a,n,U,z_{ne}\right).$$

A projection P is introduced which projects the non-equilibrium state ***Z*** onto the equilibrium subspace

(14) $$PZ\;=\;P\left(a,n,U,z_{ne}\right)\;=\;\left(a,n,U\right)\;:=\;Z^{*}$$

whose equilibrium states are marked by ${}^{*}$. A process

(15) $$Z\left(t\right)\;=\;\left(a,n,U,z_{ne}\right)\left(t\right),\;t=\mathrm{time}$$

generates by projection a trajectory on the equilibrium subspace

(16) $$PZ\left(t\right)\;\equiv \;Z^{*}\left(t\right)\;=\;\left(a,n,U\right)\left(t\right),\;z_{ne}\left(a,n,U\right)$$

which is called a reversible process, a bit strange denotation because no “process” with progress in time takes place on the equilibrium subspace. The “time” in (16) is generated by projection and represents the path parameter along the reversible process. $Z^{*}\left(t\right)$ is also denoted as the accompanying process of Z(t) [19]. Although not existing in nature, reversible processes serve as a mathematical closing of the “real” (irreversible) processes which are defined as trajectories on the non-equilibrium state space.

### 2.3. The First Law

Up to now, the internal energy was introduced in (13)${}_{1}$ as one variable of the equilibrium subspace of a thermally homogeneous Schottky system. The connexion between the time rate of the internal energy of the system and the exchange quantities through ∂G is established by the First Law

(17) $$\overset{•}{U}\;=\;\overset{•}{Q}+h\cdot {\overset{•}{n}}^{e}+\overset{•}{W}$$

which states that the internal energy *U* of the system should be conserved in isolated Schottky systems. Here, kinetic and potential energy are presupposed to be constant. The second term of the right-hand side of (17) originates from the fact that the heat exchange has to be redefined for open systems (${\overset{•}{n}}^{e}\neq 0$) [20]. Here, *h* are the molar enthalpies of the chemical components in G. The modified heat exchange which is combined with the material exchange appearing in the First Law (17) was used by R. Haase [21].

Because *U* is not defined as a state function, but rather as a state variable, $\overset{•}{U}$ is not defined as a total differential in (17). Internal mole number changes

(18) $$\overset{•}{n}\;{}^{i}\;=\;\overset{•}{n}-{\overset{•}{n}}^{e}$$

by chemical reactions are not influencing the internal energy which is also conserved in isolated systems undergoing chemical reactions [21]. How to define the internal energy in more detail can be found in [22].

In equilibrium systems, the internal energy is connected with the thermostatic equilibrium temperature by the caloric equation of state. Such a relation is missing non-equilibrium sytems, because a non-equilibrium temperature is not unequivocally defined. But fact is, that also in non-equilibria temperatures are measured, and the question arises, what is the nature of these temperatures (do not think of perfect gases).

## 3. The Second Law

### 3.1. Doubts: Non-Equilibrium Entropy

Once upon a time, Meixner wrote in 1967 [23]: ” ...The idea that an unambiguous entropy also exists in the absence of equilibrium, likewise propounded by Clausius, has been accepted almost entirely without further examination and applied with no little success. An analysis of Clausius’ work however reveals an inexactitude in the logic of his deductions which cannot be overlooked. With the aid of thermodynamic systems of the simplest kind, namely electrical networks, it can be shown that the concept of entropy in the absence of equilibrium is in fact not only questionable but that it cannot even be defined. This leads to the problem of developing a thermodynamic theory of processes .... which is disassociated from the concept of entropy in the absence of equilibrium. This latter can be achieved by applying the principle of the fundamental inequality which represents an interpretation of the Second Law of non-equilibrium thermodynamics, disassociated from the concept of entropy”.

More details concerning the “entropy-free” non-equilibrium thermodynamics which replaces the “dubious non-equilibrium entropy” by the Fundamental Inequality can be found in [24,25,26,27].

Despite of Meixner’s warning, the thermodynamic society, especially that celebrating Rational Thermodynamics uses successfully non-equilibrium entropies and also “non-equilibrium temperature” as primitive concepts. Therefore the question arises, whether there is a possibility to create a scheme for constructing non-equilibrium entropies unequivocally? Evident is, that the concept of thermostatic equilibrium temperature has to be adapted to non-equilbrium because the differential of a non-equilibrium entropy contains temperature.

### 3.2. Defining Inequalities

Consider a discrete system G and its environment $G^{□}$ which are separated from each other by a partition ∂G

**Definition** **1.** *A quantity J of G is called *balanceable*, if its time rate can be decomposed into a flux ***Ψ*** through ∂G and a production R in G.*

The flux is composed of its conductive part Φ and its convective part $\varphi {\overset{•}{n}}^{e}$. Setting

**Axiom** **1.** *The entropy is in equilibrium and in non-equilibrium a balanceable quantity.*

The equilibrium environment $G^{□}$ is presupposed to be a reservoir, that means, that the relaxation times are arbitrary high and that $G^{□}$ can be described as being always in equilibrium. Consequently, $G^{□}$ is subjected to thermostatics whose validity is presupposed. According to Axiom 1, the time rate of the entropy of the controlling reservoir is

(19) $$\overset{•}{S}\;{}^{□}\;=\;\frac{1}{T^{□}}\overset{•}{Q}\;{}^{□}+s{}^{□}\cdot \overset{•}{n}{\;}^{□e}.$$

Here, the entropy flux is a factorized decomposition into the reciprocal thermostatic temperature $T^{□}$ of the environment and the heat exchange through ∂G. Also the components of the external material exchange $\overset{•}{n}{\;}^{□e}$ are in reference to the environment. The molar entropies of the components in $G^{□}$ are $s{}^{□}$. An entropy production does not appear in (19), because $G^{□}$ is an equilibrium system and consequently, all thermostatic quantities are defined.

According to Axiom 1, a non-equilibrium entropy—which has to be defined in the sequel—has the form

(20) $$\overset{•}{S}\;=\;\frac{1}{\Theta }\overset{•}{Q}+s\cdot {\overset{•}{n}}^{e}+\Sigma .$$

Up to the exchange quantities through ∂G, $\overset{•}{Q}$ and ${\overset{•}{n}}^{e}$—which are fixed due to the inert partition according to (10)${}_{1,3}$—the temperature Θ, the molar entropies *s* and the entropy production Σ are unknown and have to be defined in the sequel. The balancebility (20) of a non-equilibrium entropy represents the first step for defining expressions which are beyond Meixner’s criticism. But first of all, Θ, *s* and Σ are only place holders in the calculation. Setting

**Axiom** **2.** *The entropies of partial systems are additive.*

The time rate of entropy of the isolated total system $G^{□}\cup G$ is according to Axiom 2

(21) $$\begin{array}{ccc} \overset{•}{S}{\;}^{tot}\;=\;\overset{•}{S}+{\overset{•}{S}}^{□} & = & \frac{1}{\Theta }\overset{•}{Q}+s\cdot {\overset{•}{n}}^{e}+\frac{1}{T^{□}}{\overset{•}{Q}}^{□}+s{}^{□}\cdot \overset{•}{n}{\;}^{□e}+\Sigma \;=\;\\ & = & \left(\frac{1}{\Theta }-\frac{1}{T^{□}}\right)\overset{•}{Q}+\left(s-s{}^{□}\right)\cdot {\overset{•}{n}}^{e}+\Sigma , \end{array}$$

by inserting the properties (10)${}_{1,3}$ of the inert ∂G.

Setting

**Axiom** **3.** *The Second Law for isolated systems—here $G^{□}\cup G$–*

(22) $$\overset{{}_{{}^{•}}}{S}{\;}^{tot}\;\geq \;0$$

results according to (21) in

(23) $$\left(\frac{1}{\Theta }-\frac{1}{T^{□}}\right)\overset{•}{Q}+\left(s-s{}^{□}\right)\cdot {\overset{•}{n}}^{e}+\Sigma \;\geq \;0.$$

Presupposing that all chemical components have the same temperature—Θ in G and $T^{□}$ in $G^{□}$—the molar entropies ***s*** can be decomposed into molar enthalpies ***h*** and chemical potentials μ [28]

(24) $$s{}^{□}\;=\;\frac{1}{T^{□}}\left(h^{□}-\mu^{□}\right),\;s\;=\;\frac{1}{\Theta }\left(h-\mu \right),$$

and (23) results in the dissipation inequality

(25) $$\left(\frac{1}{\Theta }-\frac{1}{T^{□}}\right)\overset{•}{Q}+\left(\frac{h}{\Theta }-\frac{h^{□}}{T^{□}}\right)\cdot {\overset{•}{n}}^{e}+\left(\frac{\mu^{□}}{T^{□}}-\frac{\mu }{\Theta }\right)\cdot {\overset{•}{n}}^{e}+\Sigma \;\geq \;0.$$

Setting

**Axiom** **4.** *The Second Law for entropy production—here in G–*

(26) Σ≥0.

Up to now, Θ, ***h*** and μ are place holders in the dissipation inequality (25). Their definitions have to be compatible with adiabatic processes $\left(\overset{{}_{*}}{Q}=0\right)$ and closed systems $\left({\overset{*}{n}}^{e}=0\right)$. Taking into consideration that the entropy production Σ is independent of the exchange rates which are also independent of each other, the definitions of the place holders have to be introduced in such a way that the dissipation inequality (25) is satisfied for all these special cases. This is achieved by setting

**Axiom** **5.** *In accordance with the dissipation inequality (25),* defining inequalities *are demanded for introducing the place holders *Θ*, **h** and μ of G*

(27) $$\left(\frac{1}{\Theta }-\frac{1}{T^{□}}\right)\overset{{}_{{}^{•}}}{Q}\;\overset{{}_{*}}{\geq }\;0,\;\left(\frac{h}{\Theta }-\frac{h^{□}}{T^{□}}\right)\cdot \overset{{}_{{}^{•}}}{n}\;{}^{e}\;\overset{{}_{*}}{\geq }\;0,\;\left(\frac{\mu^{□}}{T^{□}}-\frac{\mu }{\Theta }\right)\cdot \overset{{}_{{}^{•}}}{n}\;{}^{e}\;\overset{{}_{*}}{\geq }\;0,$$

*as discussed in the next section.*

## 4. Contact Quantities

First of all, the following proposition [29] is used:

(28) X·f(X)≤0(for allX∧fcontinuous atX=0)⟹f(0)=0

which is applied to (27) for an inert partition (10)${}_{1,3}$

(29) $$\left(\frac{1}{\Theta }-\frac{1}{T^{□}}\right)\overset{•}{Q}\;{}^{□}\;\leq \;0,\;\left(\frac{h}{\Theta }-\frac{h^{□}}{T^{□}}\right)\cdot \overset{•}{n}\;{}^{□e}\;\leq \;0,\;\left(\frac{\mu^{□}}{T^{□}}-\frac{\mu }{\Theta }\right)\cdot \overset{•}{n}\;{}^{□e}\;\leq \;0.$$

Without any restriction of generality, the left hand brackets in (29) can be presupposed as being continuous, if the right hand factors vanish. These factors vanish, if suitable equilibrium environments are chosen for contacting

(30) $$G_{⊙}^{□}\;⟶\;\overset{•}{Q}{\;}_{⊙}^{□}=0,\;G_{j0}^{□}\;⟶\;\overset{•}{n}{\;}_{j0}^{□e}=0.$$

$G_{⊙}^{□}$ and $G_{j0}^{□}$ are equipped with temperatures $T_{⊙}^{□}$ and $T_{0}^{□}$—the same for all ${}_{j}^{□}$-components—with molar enthalpies $h_{j0}^{□}$ and chemical potentials $\mu_{j0}^{□}$. Consequently, according to the proposition (28)

(31) $$\overset{•}{Q}{\;}_{⊙}^{□}\overset{{}_{*}}{=}0\;⟺\;\Theta =T_{⊙}^{□},\;\overset{•}{n}{\;}_{0}^{□e}\overset{{}_{*}}{=}0\;⟺\;\left(\frac{h}{\Theta }=\frac{h_{0}^{□}}{T_{0}^{□}}\right)\;\wedge \;\left(\frac{\mu }{\Theta }=\frac{\mu_{0}^{□}}{T_{0}^{□}}\right)$$

is valid. Here, (31)${}_{2}$ holds true for each chemical component. The ${}_{0}^{□}$-quantities are known and belong to the special equilibrium environments (30) which generate the vanishing right-hand side factors of (29).

Accepting the inequality (27)${}_{1}$ for defining Θ implies in connection with (10)${}_{1}$ that the conductive entropy flux through ∂G is uncontinuous

(32) $$\frac{\overset{•}{Q}}{\Theta }\;\geq \;-\frac{\overset{{}_{{}^{•}}}{Q}\;{}^{□}}{T^{□}}\;⟶\;\frac{\overset{•}{Q}}{\Theta }+\frac{\overset{{}_{{}^{•}}}{Q}\;{}^{□}}{T^{□}}\;=:\;\sigma_{Q}\;\geq \;0.$$

Analogously, the convective fluxes

(33) $$\frac{h}{\Theta }\cdot \overset{•}{n}\;{}^{e}+\frac{h^{□}}{T^{□}}\cdot \overset{•}{n}\;{}^{□e}\;=:\;\sigma_{h}\;\geq \;0,\;-\left(\frac{\mu }{\Theta }\cdot {\overset{•}{n}}^{e}+\frac{\mu^{□}}{T^{□}}\cdot \overset{•}{n}\;{}^{□e}\right)\;=:\;\sigma_{\mu }\;\geq \;0$$

generate the contact entropy productions $\sigma_{⊠}$.

### 4.1. Contact Temperature

Using (31)${}_{1}$, a non-equilibrium temperature can be introduced by measurement: Consider a non-equilibrium system G and contact it through an inert partition ∂G with the special equilibrium environment $G_{⊙}^{□}$ which generates vanishing net heat exchange $\overset{•}{Q}{\;}_{⊙}^{□}=0$, then G has by definition (31)${}_{1}$ the (non-equilibrium) contact temperature Θ which is identical with the thermostatic temperature $T_{⊙}^{□}$ of the controlling $G_{⊙}^{□}$ [30,31,32,33]. The concept of contact temperature can also be used, if the contact partition is divided into subsurfaces [16,17] Evident is, that the contact temperature depends on the design of the partition ∂G. This influence vanishes in equilibrium, and the contact temperature changes into the thermostatic temperature.

There are a lot of suggestions for non-equilibrium temperatures [34]. But the problem is to find a thermometer for the proposed non-equilibrium temperature, otherwise it presents only a quantity of calculation [35]. The contact temperature is defined by such a thermometer: this is $G_{⊙}^{□}$ having the thermostatic temperature $T_{⊙}^{□}=:\Theta$. In more detail:

**Definition** **2.** *The system’s contact temperature is that thermostatic temperature of the system’s equilibrium environment for which the net heat exchange between the system and this environment through an inert partition vanishes by change of sign.*

As easily to demonstrate, contact temperature Θ and the internal energy *U* are independent of each other. For this purpose, a rigid inert partition ∂G ($\overset{•}{a}\equiv 0$) is chosen which is impervious to matter $\left({\overset{•}{n}}^{e}\equiv 0\right)$ and a time-dependent environment temperature $T^{□}\left(t\right)$ which is always set equal to the value of the momentary contact temperature Θ(t) of G:

(34) $$T^{□}\left(t\right)\overset{{}_{*}}{=}\Theta \left(t\right)\;⟶\;\overset{•}{Q}\;{}^{□}=-\overset{•}{Q}\;=\;0\;⟶\;\overset{•}{U}\;=\;0$$

according to (17). Because Θ is time-dependent and *U* is constant, totally different from thermostatics, both quantities are independent of each other.

The contact temperature is useful for defining efficiencies which are smaller than the Carnot efficiency [15,36]. Consequently, this non-equilibrium efficiency represents a more realistic quantity for process evaluation.

### 4.2. Non-Equilibrium Molar Enthalpies and Chemical Potentials

Using (31)${}_{2}$, a non-equilibrium molar entropy can be introduced by measurement: Consider a non-equilibrium Schottky system G and contact it through an inert partition ∂G with the special equilibrium environment $G_{nj}^{□}$ which generates vanishing net material exchange of the ${}_{j}^{□}$-component $\overset{•}{n_{j}}\;{\;}^{□e}=0$, then G has by definition (31)${}_{2}$ the non-equilibrium molar enthalpy (non-equilibrium chemical potential)

(35) $$h_{j}\;=\;\frac{\Theta }{T_{0}^{□}}h_{j0}^{□}\;⟶\;h\;=\;\frac{T_{⊙}^{□}}{T_{0}^{□}}h_{0}^{□},\;(\text{and analogously}\;\mu \;=\;\frac{T_{⊙}^{□}}{T_{0}^{□}}\mu_{0}^{□})$$

which is identical with the thermostatic molar enthalpy $h_{j0}^{□}$ of the controlling environment $G_{nj}^{□}$, if besides the vanishing material exchange $\overset{•}{n_{j}}\;{\;}^{□e}=0$ also the heat exchange vanishes

(36) $$T_{0}^{□}≐T_{⊙}^{□}:\;\left(\overset{{}_{{}^{•}}}{n}\;{\;}^{□e}=0\right)\;\wedge \;\left(\overset{{}_{{}^{•}}}{Q}{\;}_{⊙}^{□}=0\right)\;⟶\;h=h_{0}^{□},\;\left(\text{and analogously}\;\mu =\mu_{0}^{□}\right).$$

Evident is, that different measuring devices (different controlling equilibrium environments) are necessary for defining the contact temperature Θ, the non-equilibrium molar enthalpies $h_{j}$ and chemical potentials $\mu_{j}$ of the chemical components in G.

### 4.3. Non-Equilibrium Molar Entropies

According to (24)${}_{2}$, (35) and (36)${}_{1}$, the non-equilibrium molar entropy is

(37) $$s\;=\;\frac{1}{\Theta }\left(h-\mu \right)\;=\;\frac{1}{T_{0}^{□}}\left(h_{0}^{□}-\mu_{0}^{□}\right)\;=\;s_{0}^{□}.$$

The non-equilibrium molar entropy is defined by the thermostatic molar entropy of that equilibrium environment that generates vanishing material exchange.

Here is the synopsis of the contact quantities, their defining quantities and the corresponding controlling equilibrium environments:

(38) $$\begin{array}{ccc} G & \;⟶\; & \Theta ,h,\mu ,s\;\text{non-equilibrium} \end{array}$$

(39) $$\begin{array}{ccc} G^{□} & \;⟶\; & T^{□},h^{□},\mu^{□},s^{□}{\;}^{□}\text{equilibrium environment} \end{array}$$

(40) $$\begin{array}{ccc} G_{⊙}^{□} & \;⟶\; & T_{⊙}^{□}\;\text{vanishing heat exchange} \end{array}$$

(41) $$\begin{array}{ccc} G_{nj}^{□} & \;⟶\; & T_{0}^{□},h_{j0}^{□},\mu_{j0}^{□},s_{j0}^{□}\;\text{vanishing material exchange} \end{array}$$

The quantities ${⊠}_{⊙,0}^{□}$ marked by ⊙ or zero are given thermostatic quantities of several equilibrium environments. The resulting contact quantities of (38) are determined by the following equations: (31)${}_{1,2}$ and (37). The construction of a non-equilibrium entropy rate can now go on.

## 5. Verification: Non-Equilibrium Entropy

Starting with the preliminary shape of the entropy rate (20), the contact temperature Θ is now defined by (31)${}_{1}$ and the non-equilibrium molar entropy by (37). The shape of the entropy production Σ is still missing. Beyond that, the entropy rate (20) is up to now no time derivative of a state function of the Schottky system G (oldfashioned: no total differential of something), but only a time rate along a process. Consequently, a suitable non-equilibrium state space has to be specified in (13)${}_{2}$ which allows to generate a non-equilibrium entropy as a state function on it.

### 5.1. A Non-Equilibrium State Space

Because the equilibrium subspace (13)${}_{1}$ is spanned by the work variables ***a***, the mole numbers ***n*** and the internal energy *U*, these state variables appear also in the non-equilibrium state space. If the First Law (17) and (24)${}_{2}$ are inserted, (20) results in

(42) $$\overset{•}{S}\;=\;\frac{1}{\Theta }\left(\overset{{}_{{}^{•}}}{U}-h\cdot \overset{{}_{{}^{•}}}{n}\;{}^{e}-A\cdot \overset{{}_{{}^{•}}}{a}+h\cdot \overset{{}_{{}^{•}}}{n}\;{}^{e}-\mu \cdot \overset{{}_{{}^{•}}}{n}\;{}^{e}\right)+\Sigma .$$

Because the external mole number rates ${\overset{•}{n}}^{e}$ are no state variables, but the mole numbers themselves are included in the equilibrium subspace (13)${}_{1}$ according to the Zeroth Law, the missing term for generating the mole numbers in (42) is hidden in the entropy production

(43) $$\Sigma \;=\;-\frac{1}{\Theta }\mu \cdot \overset{•}{n}\;{}^{i}+\Sigma^{0},$$

and taking (18) into account, (42) results in

(44) $$\overset{•}{S}\;=\;\frac{1}{\Theta }\left(\overset{{}_{{}^{•}}}{U}-A\cdot \overset{{}_{{}^{•}}}{a}-\mu \cdot \overset{{}_{{}^{•}}}{n}\right)+\Sigma^{0}.$$

Because the bracket in (44) contains only equilibrium variables, the non-equilibrium state variables appear in the entropy production $\Sigma^{0}$. Because the contact temperature is independent of the internal energy, it represents an additional variable which is included in $\Sigma^{0}$. The choice of further non-equilibrium variables depends on the system in consideration. Here, *internal variables* ξ are chosen because they allow a great flexibility in describing non-equilibria [37,38]. Consequently, the created non-equilibrium state space is

(45) Z=(a,n,U,Θ,ξ)

(this is an example, other state spaces are of course possible). The entropy rate (44) and the entropy production with regard to the non-equilibrium variables Θ and ξ become

(46) $$\overset{•}{S}\left(Z\right)\;=\;\frac{1}{\Theta }\left(\overset{{}_{{}^{•}}}{U}-A\cdot \overset{{}_{{}^{•}}}{a}-\mu \cdot \overset{{}_{{}^{•}}}{n}\right)+\alpha \overset{•}{\Theta }+\beta \cdot \overset{•}{\xi }\;⟶\;\Sigma^{0}=\alpha \overset{•}{\Theta }+\beta \cdot \overset{•}{\xi }\;\geq \;0.$$

The time rate along a process on a state space does not necessarily belong to a state function: two additional requirements must be satisfied, firstly the embedding theorem which guarantees that the so constructed non-equilibrium entropy time rate is in accordance with the presupposed equilibrium entropy, and secondly the adiabatical uniqueness which enforces that the time rate of the non-equilibrium entropy is a total differential of the state space variables.

### 5.2. Embedding Theorem

The time rate of the non-equilibrium entropy has to be in accordance with the—as known presupposed—equilibrium entropy: the non-equilibrium entropy rate integrated along an irreversible process T starting and ending in equilibrium states—$A_{eq}$ and $B_{eq}$—has the same value as the equilibrium entropy difference between these two equilibrium states calculated along the corresponding accompanying process R (16)

(47) $$T\int_{Aeq}^{Beq}\overset{•}{S}\left(Z\right)dt\;=\;R\int_{Aeq}^{Beq}\overset{•}{S}\left(P(Z)\right)dt\;=\;S\left(B_{eq}\right)-S\left(A_{eq}\right).$$

Taking the projection (16) and the entropy production (46)${}_{2}$ into account, (47) results in

(48) $$\begin{array}{ccc} 0\; & = & T_{R}\int_{Aeq}^{Beq}\left(\overset{{}_{{}^{•}}}{S}\left(Z\right)-\overset{{}_{{}^{•}}}{S}\left(P\left(Z\right)\right)\right)dt\;\geq \;\\ & \geq & T_{R}\int_{Aeq}^{Beq}\left[\left(\frac{1}{\Theta }-\frac{1}{T^{*}}\right)\overset{{}_{{}^{•}}}{U}-\left(\frac{A}{\Theta }-\frac{A^{*}}{T^{*}}\right)\cdot \overset{{}_{{}^{•}}}{a}-\left(\frac{\mu }{\Theta }-\frac{\mu^{*}}{T^{*}}\right)\cdot \overset{{}_{{}^{•}}}{n}\right]dt. \end{array}$$

This inequality reminds of the “Fundamentale Ungleichung” [25] of the “Entropiefreie Thermodynamik” [26,39] because an entropy does not appear in (48). Of course, fifty years later the formal background is different: first of all, the realization that the introduction of a non-equilibrium entropy also requires an introduction of a well defined non-equilibrium temperature, that reversible accompanying processes arise from projections on the equilibrium subspace and that a non-equilibrium large state space is necessary for defining a non-equilibrium entropy as a state function.

Consider a cyclic process which at least contains one equilibrium state $A_{eq}$, (47) results in

(49) $$B_{eq}⪸A_{eq}:{\;}^{\left(A_{eq}\right)}\;\;\oint \overset{•}{S}\left(Z\right)dt\;=\;0,$$

a relation which does not enable the entropy rate to be a total differential on the state space because this special cyclic processes contains at least one equilibrium state. A generalization is made in the next section.

### 5.3. Adiabatical Uniqueness

Consider an arbitrary, but fixed non-equilibrium state *B* and a process family whose processes T all start at different arbitrary equilibrium states $A_{eq}$ and end in *B*. Subsequently, an adiabatic process takes place starting from *B* and ending in an equilibrium state $C_{eq}$

(50) $$\begin{array}{ccc} T:\;A_{eq} & ⟶ & B,\;\mathrm{different}\;A_{eq},\mathrm{arbitrary}\;T,\mathrm{fixed}\;B, \end{array}$$

(51) $$\begin{array}{ccc} A:\;B & ⟶ & C_{eq},\;\mathrm{fixed}\;B,\mathrm{adiabatic}\;A. \end{array}$$

The entropy change along these processes is according to the embedding theorem

(52) $$\begin{array}{cc} & T\int_{A_{eq}}^{B}\overset{•}{S}dt+A\int_{B}^{C_{eq}}\overset{•}{S}dt\;=\;S_{eq}^{C}-S_{eq}^{A}, \end{array}$$

(53) $$\begin{array}{cc} & T\int_{A_{eq}}^{B}\overset{•}{S}dt\;=\;S^{B}\left(A_{eq},T\right)-S_{eq}^{A}. \end{array}$$

The non-equilibrium entropy $S^{B}$ may depend on T and its starting state $A_{eq}$. Inserting (53) into (52) results in

(54) $$S^{B}\left(A_{eq},T\right)\;=\;S_{eq}^{C}-A\int_{B}^{C_{eq}}\overset{•}{S}dt.$$

If the final equilibrium state $C_{eq}$ in which the adiabatic process ends does not depend on different $\left(A_{eq},T\right)$, also the entropy $S^{B}$ of the fixed *B* does not depend on them and the left-hand side of (54) represents a process independent non-equilibrium entropy (not only a rate) of arbitrary chosen non-equilibrium states *B* whose value is given by the right-hand side of (54).

This uniqueness is satisfied in phenomenological, but not in stochastic thermodynamics [40]. In more detail, the condition runs as follows [15]

**Definition** **3.** *A Schottky system is called* adiabatically unique*, if for each arbitrary, but fixed non-equilibrium state B after isolation of the system, the relaxation process ends always in the same final equilibrium state, independently of how the process into B was performed.*

Consequently, a non-equilibrium entropy of Schottky systems

(55) $$S^{B}\left(Z\right)\;=\;S_{eq}^{C}-A\int_{B}^{C_{eq}}\overset{•}{S}dt$$

can always be defined as a state function on a non-equilibrium state space, if the system is adiabatically unique. This well founded definition is evidently more than a “primitive concept” to which Meixner objected. Also along a non-equilibrium process, the entropy time rate inequality

(56) $$\overset{•}{S}\;\geq \;\frac{1}{\Theta }\overset{•}{Q}+s\cdot {\overset{•}{n}}^{e}$$

is in this case valid according to (20) and (26). Consequently, one of the conditions which Meixner missed when he stated [23] “there is no entropy along an irreversible process” is valid and results in a generalization of Clausius inequality

(57) $$\oint \overset{•}{S}dt\;=\;0\;\geq \;\oint \left(\frac{1}{\Theta }\overset{{}_{{}^{•}}}{Q}+s\cdot \overset{{}_{{}^{•}}}{n}\;{}^{e}\right)dt$$

in which no quantities of the controlling environment appears. The original Clausius inequality for open systems results from use of the defining inequalities (27)

(58) $$\begin{array}{c} 0\;\geq \;\oint \left(\frac{1}{\Theta }\overset{{}_{{}^{•}}}{Q}+s\cdot \overset{{}_{{}^{•}}}{n}\;{}^{e}\right)dt\;\geq \;\oint \left(\frac{1}{T^{□}}\overset{{}_{{}^{•}}}{Q}+s^{□}\cdot \overset{{}_{{}^{•}}}{n}\;{}^{e}\right)dt\;=\;SL, \end{array}$$

(59) $$\begin{array}{c} \oint \left[\left(\frac{1}{\Theta }-\frac{1}{T^{□}}\right)\overset{{}_{{}^{•}}}{Q}+\left(s-s^{□}\right)\cdot \overset{{}_{{}^{•}}}{n}\;{}^{e}\right]dt\;\geq \;0.\; \end{array}$$

The cyclic path integral runs on the non-equilibrium state space. Consequently, the four questions posed at the beginning of the paper are answered. Especially, the reasoning—defining inequalities, non-equilibrium entropy, extended and ordinary Clausius inequality—elucidates how to answer the questions. Reversible or better accompanying processes are by projection generated mathematical pathes on the equilibrium subspace. Unusual is, that internal energy and contact temperature are independent state variables, the first an equilibrium variable and the second a non-equilibrium one. Conclutions resulting from this fact are discussed in the next section.

### 5.4. The Integrability Conditions

If a Schottky system G is adiabatically unique, a non-equilibrium entropy exists according to (55)

(60) S=S(U,a,n,Θ,ξ),

and the path integrals on the non-equilibrium state space over the entropy rate between two states are path independent. Because (60) is a state function, from (46)${}_{1}$ follows for the partial derivatives of the entropy

(61) $$\begin{array}{c} \frac{\partial S}{\partial U}\;=\;\frac{1}{\Theta },\;\frac{\partial S}{\partial a}\;=\;-\frac{A}{\Theta },\;\frac{\partial S}{\partial n}\;=\;-\frac{\mu }{\Theta }, \end{array}$$

(62) $$\begin{array}{c} \frac{\partial S}{\partial \Theta }\;=\;\alpha ,\;\frac{\partial S}{\partial \xi }\;=\;\beta . \end{array}$$

Because Θ and *U* are independent of each other according to (34), we can integrate (61)${}_{1}$ immediately

(63) $$S\left(U,a,n,\Theta ,\xi \right)\;=\;\frac{1}{\Theta }U+K\left(a,n,\Theta ,\xi \right).$$

Consequently, the non-equilibrium entropy is a linear function of the internal energy. Here

(64) U-ΘS=-ΘK=:F(a,n,Θ,ξ)

is the free energy *F* of G.

Because of (12) and (14), in equilibrium is valid

(65) $$S^{*}\;=\;\frac{1}{T^{*}}U-\frac{F}{T^{*}}\left(a,n,\Theta (U,a,n),\xi (U,a,n)\right),$$

an expression which is in general non-linear in *U*. Consequently, the equilibrium entropy $S^{*}$ is in contrast to the non-equilibrium entropy (63) non-linear in *U*. From the integrability conditions (61) and (62) follows that except of α all constitutive equations do not depend on the internal energy *U* in non-equilibrium:

(66) $$\begin{array}{ccc} \frac{\partial }{\partial a}\frac{\partial S}{\partial U} & = & 0\;⟹\;\frac{\partial A}{\partial U}\;=\;0, \end{array}$$

(67) $$\begin{array}{ccc} \frac{\partial }{\partial n}\frac{\partial S}{\partial U} & = & 0\;⟹\;\frac{\partial \mu }{\partial U}\;=\;0, \end{array}$$

(68) $$\begin{array}{ccc} \frac{\partial }{\partial \xi }\frac{\partial S}{\partial U} & = & 0\;⟹\;\frac{\partial \beta }{\partial U}\;=\;0, \end{array}$$

(69) $$\begin{array}{ccc} \frac{\partial }{\partial \Theta }\frac{\partial S}{\partial U} & = & -\frac{1}{\Theta^{2}}\;=\;\frac{\partial \alpha }{\partial U}\;⟶\;\alpha \;=\;-\frac{U}{\Theta^{2}}. \end{array}$$

Apart from these specialities caused by the independence of the contact temperature from the internal energy, the formal structure of phenomenological non-equilibrium thermodynamics is except of the entropy production very similar to thermostatics.

## 6. Summary

Meixner’s historical remark [23] “... it can be shown that the concept of entropy in the absence of equilibrium is in fact not only questionable but that it cannot even be defined" was the starting-point of a deliberation, if there is a possibility to introduce non-equilibrium entropies which are better founded than a primitive concept. Here, steps are done for a basic substantiation of non-equilibrium entropies for Schottky systems (concerning field formulation see [41,42]). The chain of reasoning is as follows:

- Introduce a large state space [18] which is composed of an equilibrium subspace and a non-equilibrium part
- The variables of the equilibrium subspace are determined by the Zeroth Law: work variables, mole numbers and internal energy
- Processes are trajectories on the state space
- Reversible processes are projections of non-equilibrium processes onto the equilibrium subspace. An accompanying process [19] with time as a path parameter is generated by projection of the corresponding non-equilibrium process onto the equilibrium subspace
- The time rate of the internal energy is introduced by the First Law
- The entropy is in equilibrium and in non-equilibrium a balanceable quantity
- The entropies of partial systems are additive
- Introduction of the Second Law for isolated systems and entropy productions [14]
- The defining inequalities for contact temperature, non-equilibrium molar enthalpies and chemical potentials resulting in the non-equilibrium molar entropy
- The embedding theorem enforcing compatibility of a non-equilibrium entropy with the equilibrium one
- Adiabatic uniqueness guaranteeing that the non-equilibrium entropy is a state function on the non-equilibrium state space.

These items make it possible to generate a non-equilibrium entropy as a state function. Beyond that, the Clausius inequality for open systems (58)${}_{2}$ follows including thermostatic temperature and equilibrium molar entropy of the system’s controlling environment. Additionally, the four questions of the introduction are answered.

Fifty years ago, the thermodynamical society was not aware that the above mentioned items are necessary to get rid of the primitive concept of a non-equilibrium entropy. Thus, it is evident that a more axiomatically oriented thermodynamicist was endeavoured at that time to avoid the use of a non-equilibrium entropy.

## 7. Closure

Non-equilibrium open Schottky systems are characterized by contact quantities whose definitions require inert partitions. If contact quantities seem to be too artificial, the non-equilibrium Schottky system can be approximatively replaced by an equilibrium one which is described by the accompaying process (16) of the Schottky system resulting in the contact of two equilibrium systems. This kind of description is called endoreversible thermodynamics. The non-equilibrium variable contact temperature becomes dependent on the internal energy and the other equilibrium variables, known as caloric equation of state. The entropy productions in the Schottky system and its environment vanish, but the contact entropy productions remain because the contact problem is unchanged. The non-equilibrium contact quantities of the Schottky system are replaced by the equilibrium quantities of the accompanying process resulting in the contact entropy productions (32) and (33). Endoreversible thermodynamics is analogous to the hypothesis of local equilibrium in field theories of thermodynamics. 
